# Author Correction: A CAR RNA FISH assay to study functional and spatial heterogeneity of chimeric antigen receptor T cells in tissue

**DOI:** 10.1038/s41598-021-96979-0

**Published:** 2021-08-30

**Authors:** Karsten Eichholz, Alvason Zhenhua Li, Kurt Diem, Michael Claus Jensen, Jia Zhu, Lawrence Corey

**Affiliations:** 1 Vaccine and Infectious Disease Division, Fred Hutchinson Cancer Research Center, 1100 Fairview Ave N, MS E3‑300, Seattle, WA 98190 USA; 2 Department of Laboratory Medicine, University of Washington, Seattle, WA USA; 3 Clinical Research Division, Fred Hutchinson Cancer Research Center (FHCRC), Seattle, WA USA; 4 Ben Towne Center for Childhood Cancer Research, Seattle Children’s Research Institute, Seattle, WA USA; 5 Department of Medicine, University of Washington, Seattle, WA USA

Correction to: *Scientific Reports* 10.1038/s41598-021-92196-x, published online 21 June 2021

The original version of this Article contained errors in Figure 2 where a previous version of the image was published. As a result, panels F and G were incorrectly included.

The original Figure 2 and accompanying legend appears below.

**Figure 2:**
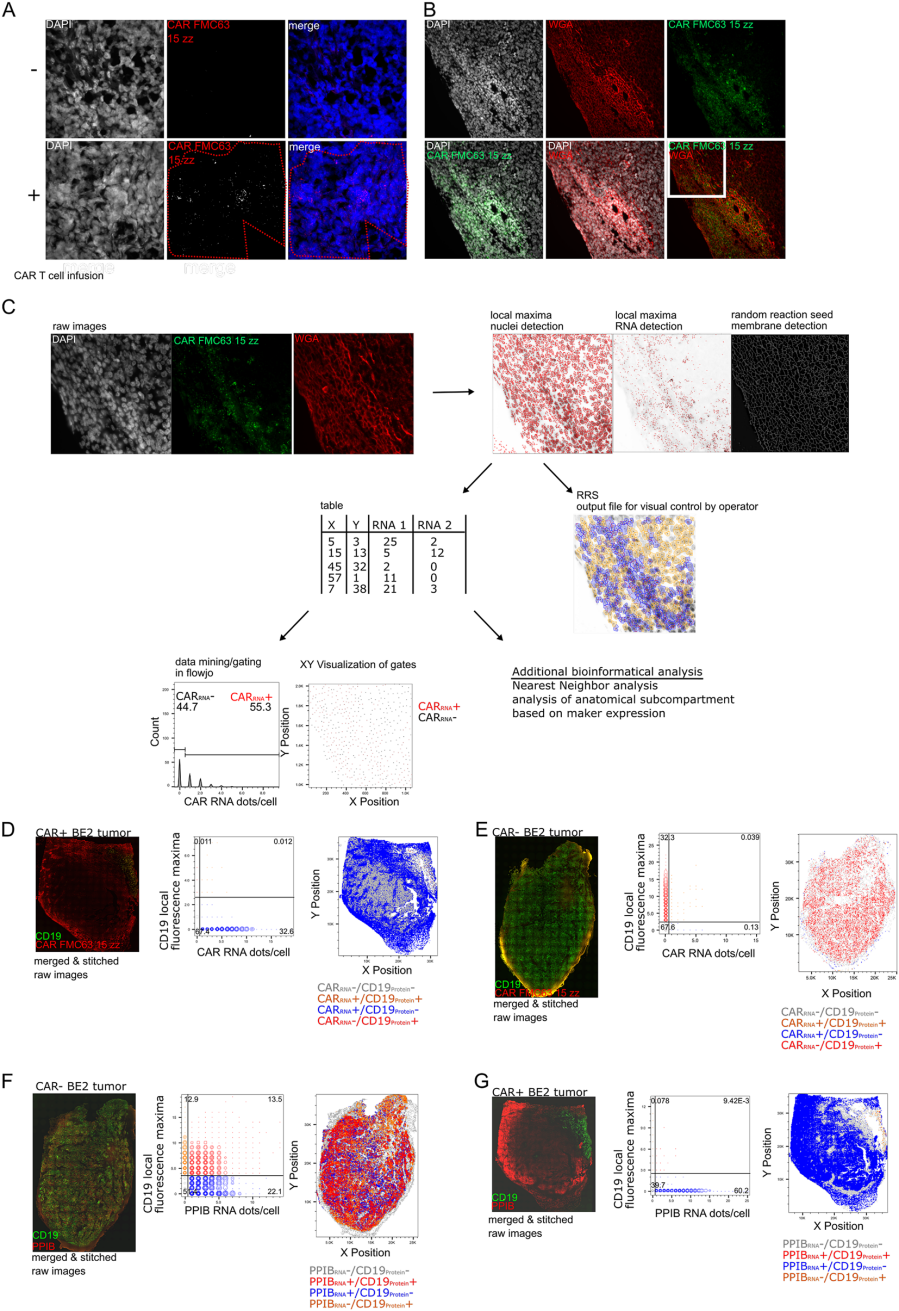
In situ detection of anti-CD19-CAR T cells in mouse xenografts of NSG mice after anti-CD19 CAR T cell infusion. FFPE-BE2 neuroblastoma xenografts with ectopic expression of truncated CD19 treated with anti-CD19 CAR T cell for 7 days or without CAR T cells was used for the CAR RNA FISH in situ validation. Staining of CAR+ and CAR− FFPE-BE2 tumor tissue sections with CAR FMC63 15zz probes (red). The red dotted line denotes an area that showed specific CAR RNA FISH staining (**A**). Staining of CAR+ BE2 tumor with CAR FMC63 15zz probes and Texas Red-conjugated wheat germ agglutinin improves cell segmentation (**B**). RRS analysis of multicolor raw images includes nuclei and CAR RNA detection as well RRS detection of plasma membrane staining. The algorithm generates a table internally containing the RNA dots/cell and the x–y position of every detected cell, which can be exported for data mining in Flowjo to assess and visualizedifferential RNA expression and locations of different cell types. These data can be further used for additional bioinformatical analysis. The example picture derives from the area framed in white in B (**C**). Whole tissue overview images show the distribution of the CAR RNA and CD19 IHC staining combined with WGA and DAPI (not shown) and the whole tissue RRS analysis of CAR+ (n = 3) (**D**) and CAR− (n = 3) (**E**) tumor section.

The original Article has been corrected.

