# Supplementary material for: A CAR RNA FISH assay to study functional and spatial heterogeneity of chimeric antigen receptor T cells in tissue
Source: Sci Rep. 2021 Jun 21;11:12921. doi: 10.1038/s41598-021-92196-x (PMC8217486; doi:10.1038/s41598-021-92196-x)
Supplement: Supplementary file 1 — Supplementary Information 1. [file 41598_2021_92196_MOESM1_ESM.docx]

**Supplementary Figure 1: CAR probe table, principle of RNA FISH.**

Three zz probes sets were designed for detection of anti-CD19 CAR T cells. The probes bind to the scFv, the intergeneic regions between the signaling domains and some probes sets to the 3’ untranslated region of the CAR mRNA, which constitute nucleic acid sequences that cannot be found in non-genetically engineered organisms (A).

**Supplementary Figure 2: purity of primary CAR T cell culture after expansion.**

Anti-CD19 CD8 CAR T cells were cultured with γ-irradiated CD19+ TM-LCL cells to stimulate expansion. On day 12 of cell culture, CAR T cell purity was assessed by flow cytometry for the cell surface markers CD19-BV786, EGFRt-PE and CD8-FITC (n=1). These cells were processed for CAR RNA FISH and used for the *in vitro* validation.

**Supplementary Figure 3: CAR RNA FISH detects CAR RNA mainly extranuclear.**

CAR Jurkat cells were stained with CARprobes and nuclear counterstain to exemplify subcellular localization of CAR RNA. The CAR RNA is mainly detected extranuclear or on the outline of the nuclear counterstain.

**Supplementary Figure 4: presence and expression of CAR sequence and wPRE in mouse xenografts.**

ddPCR analysis for the presence of the CAR signaling domain (CD8CD3) and the 3’UTR containing the wPRE sequence of genomic DNA in xenograft tissue section from CAR T cell-infused and control mice. (n=2). ddPCR primers and probes were directed against the CD3 CD8 region and wPRE portion to test for CAR and CD19 lentivirus-derived expression cassettes.

**Supplementary Figure 5: CAR RNA FISH in situ validation.**

Specificity of CAR RNA FISH in situ staining was further validated with RNA FISH staining for housekeeping gene *PPIB* (A) and bacterial gene *DapB* (B) CAR RNA FISH protocol without the addition of CAR FMZ63 15zz probes (C) or after RNAse treatment (D) (n=3).

**Supplementary Figure 6: Comparison of manual and RRS cell counting**

We chose 10 images from the CAR+ BE2 tumor dataset that are exemplary for images without artifacts or that show different imaging artifacts like strong membrane staining or weak nuclei staining (A). Total cell numbers were counted manually by three operators or with the RRS algorithm using the nuclei and membrane WGA staining (B). Correlation of the number of cells counted/images by the three operators and the RRS method (C). The counting time required/image is shown in (D).

**Supplementary Figure 7: Low effector gene expression in CAR T cells that are located further in the CD19low area.**

CAR T cell functional phenotyping in a BE2 neuroblastoma xenograft was done by IHC for tumor marker CD19 and CAR RNA FISH in combination with either *GZMB* (n=5) or *IFNγ* RNA FISH (n=5). Confocal images of the combination of CAR RNA and CD19 with either *GZMB* RNA (A) or *IFNγ* RNA (B) in a representative area in the CD19low area. Exemplary wide field images that were used to create the overview thumbnail images in Figure 4 image of CAR RNA, CD19 with either *GZMB* RNA (C) or *IFNγ* RNA (D).

**Supplementary Figure 8: RRS analysis CD19, CAR and *GZMB* expression of tissue sections from a BE2 tumor.**

CAR T cell functional phenotyping on consecutive section of a BE2 neuroblastoma xenograft was done by counterstaining for nuclei and membranes (not shown) as well as IHC for tumor marker CD19 and CAR RNA FISH in combination with *GZMB* RNA FISH. The images were analyzed by RRS and plotted in FlowJo (n=4).

**Supplementary Figure 9: RRS analysis CD19, CAR and *IFNγ* expression of tissue sections from a BE2 tumor.**

CAR T cell functional phenotyping on consecutive section of a BE2 neuroblastoma xenograft was done by counterstaining for nuclei and membranes (not shown) as well as IHC for tumor marker CD19 and CAR RNA FISH in combination with *IFNγ* RNA FISH. The images were analyzed by RRS and plotted in FlowJo(n=4).

**Supplementary Table 1: CAR RNA FISH probes and respective catalog number.**

| Probe name | Catalog Number |
| --- | --- |
| FMC63 15ZZ | 515321 |
| FMC63 26ZZ | 513551 |
| DapB-C1 | 310043 |
| Hs-PPIB-C2 | 313901-C2 |
| Hs-PPIB-C1 | 313901-C1 |
| Hs-GZMB | 445971 |
| Hs-GZMB-C2 | 445971-C2 |
| Hs-CD8A-C3 | 560391-C3 |
| Hs-CD4-C2 | 605601-C2 |
| Hs- IFNγ-C1 | 310501-C1 |
| Hs- IFNγ-C2 | 310501-C2 |
