# Supplementary material for: A CAR RNA FISH assay to study functional and spatial heterogeneity of chimeric antigen receptor T cells in tissue
Source: Sci Rep. 2021 Jun 21;11:12921. doi: 10.1038/s41598-021-92196-x (PMC8217486; doi:10.1038/s41598-021-92196-x)
Supplement: Supplementary file 2 — Supplementary Information 2. [file 41598_2021_92196_MOESM2_ESM.pdf]

	total zz	scFv	CAR core	3'UTR	target tissue/species
FMC63 15zz	15	11	4	0	human and mouse tissue containing different lentiviral constructs
FMC63 27zz	26	11	4	12	human and mouse tissue with only CAR T cells present

Supplementary Figure 1

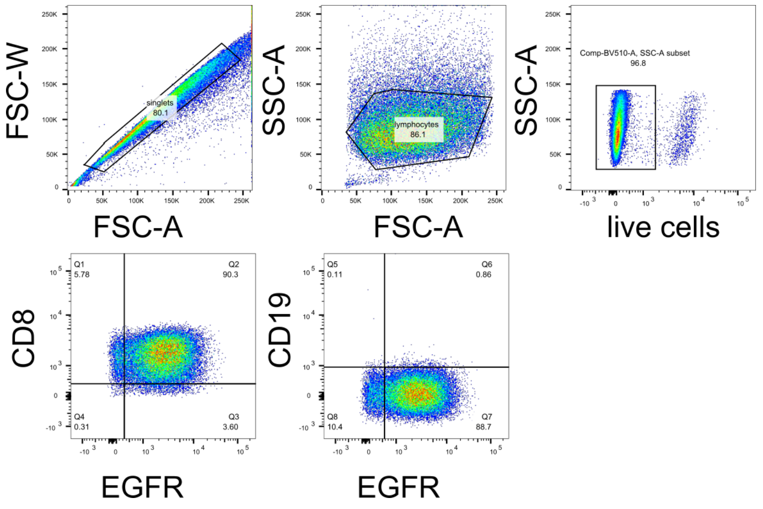

Supplementary Figure 2

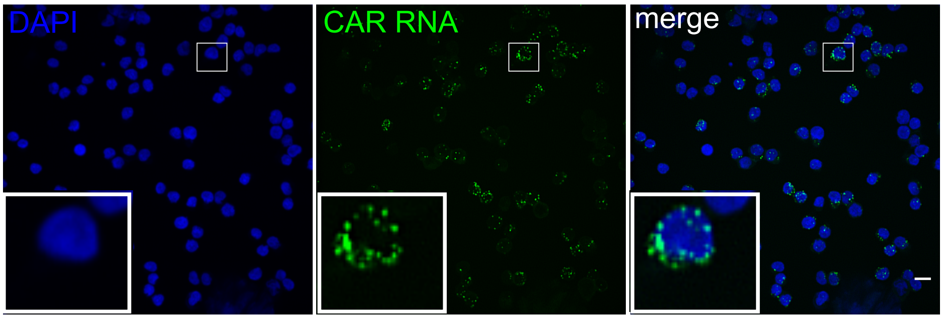

Supplementary Figure 3

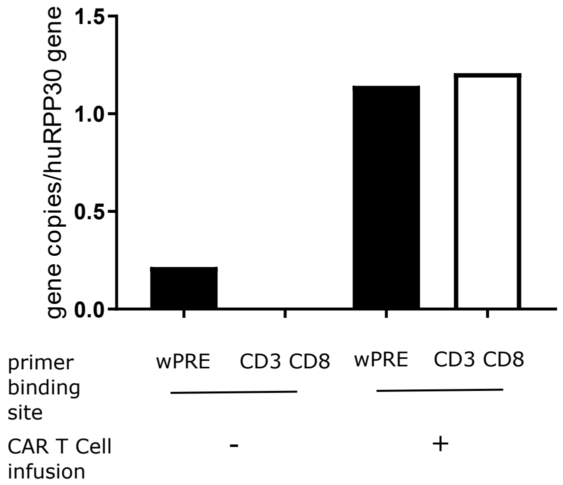

Supplementary Figure 4

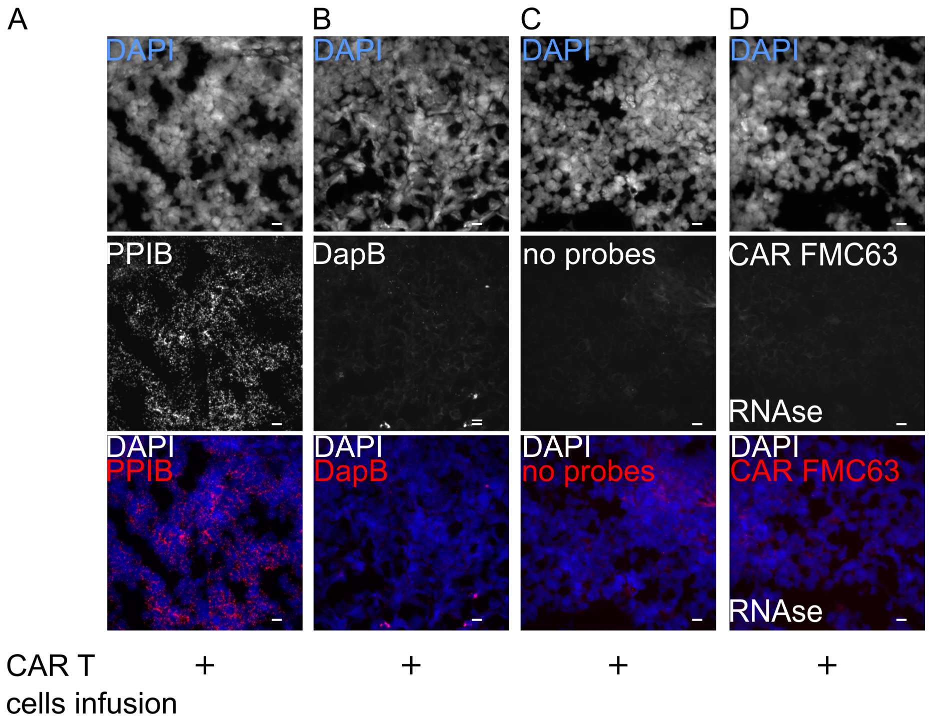

Supplementary Figure 5

A

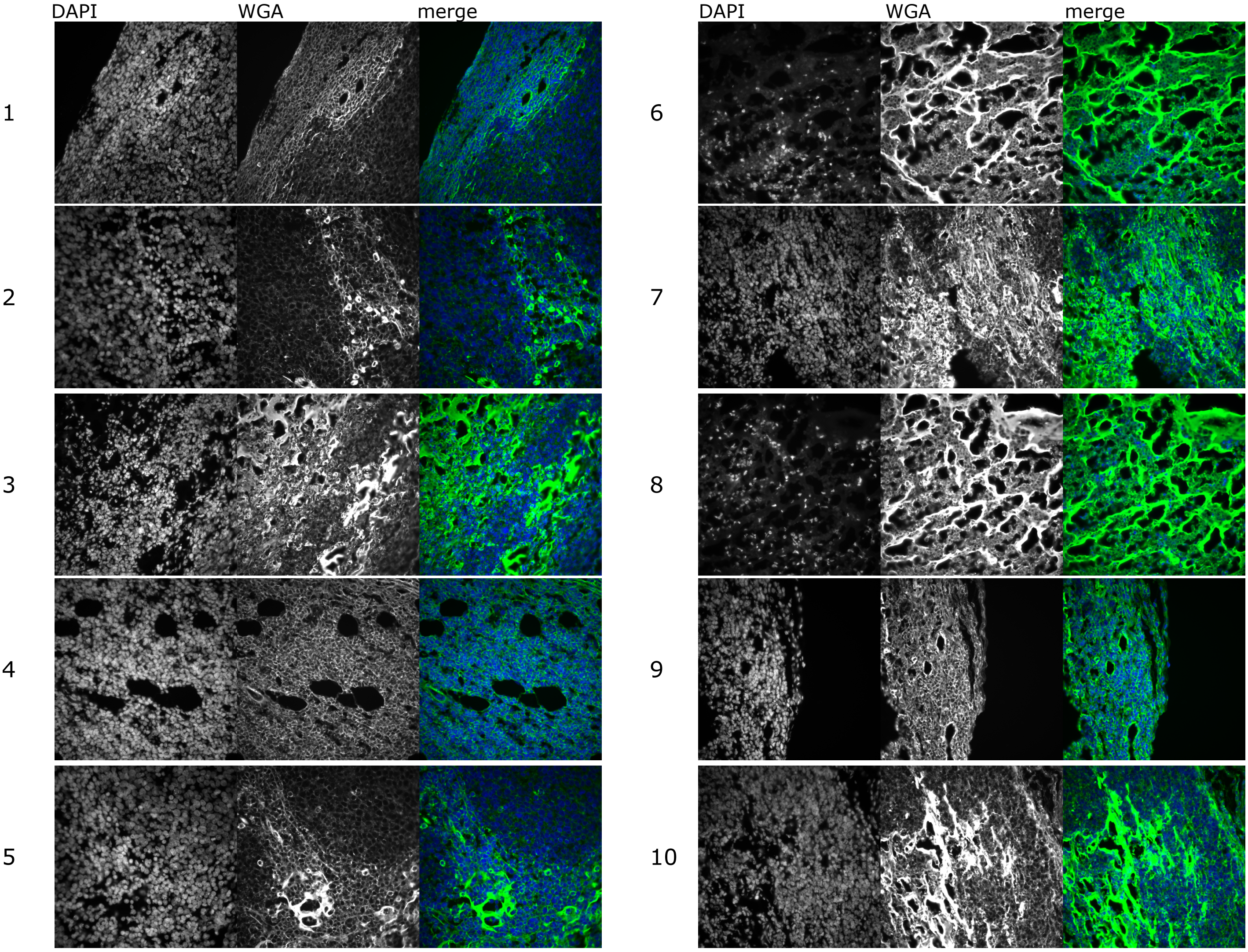

B

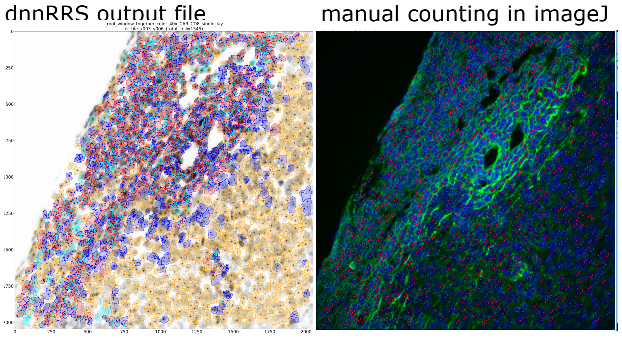

C

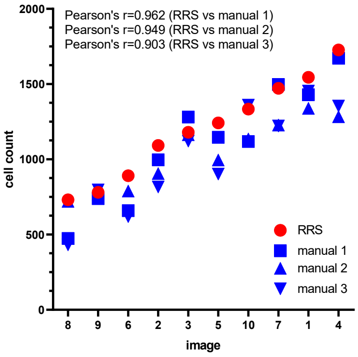

D

image	dnnRRS (h:mm:ss)	manual 1 (h:mm:ss)	manual 2 (h:mm:ss)	manual 3 (h:mm:ss)
1	0:01:41	0:14:50	0:12:26	0:41:19
2	0:01:41	0:08:49	0:07:29	0:28:44
3	0:01:43	0:11:27	0:10:06	0:29:55
4	0:01:27	0:14:56	0:11:25	0:23:34
5	0:01:49	0:10:16	0:09:16	0:13:40
6	0:01:32	0:15:01	0:07:57	0:11:37
7	0:01:38	0:14:02	0:10:47	0:18:37
8	0:00:58	0:10:56	0:07:09	0:25:23
9	0:01:07	0:12:35	0:08:09	0:15:41
10	0:01:06	0:20:46	0:12:45	0:26:03
avergage time/image	0:01:28		0:15:31	
SD	0:00:18		0:08:02	

Supplementary Figure 6

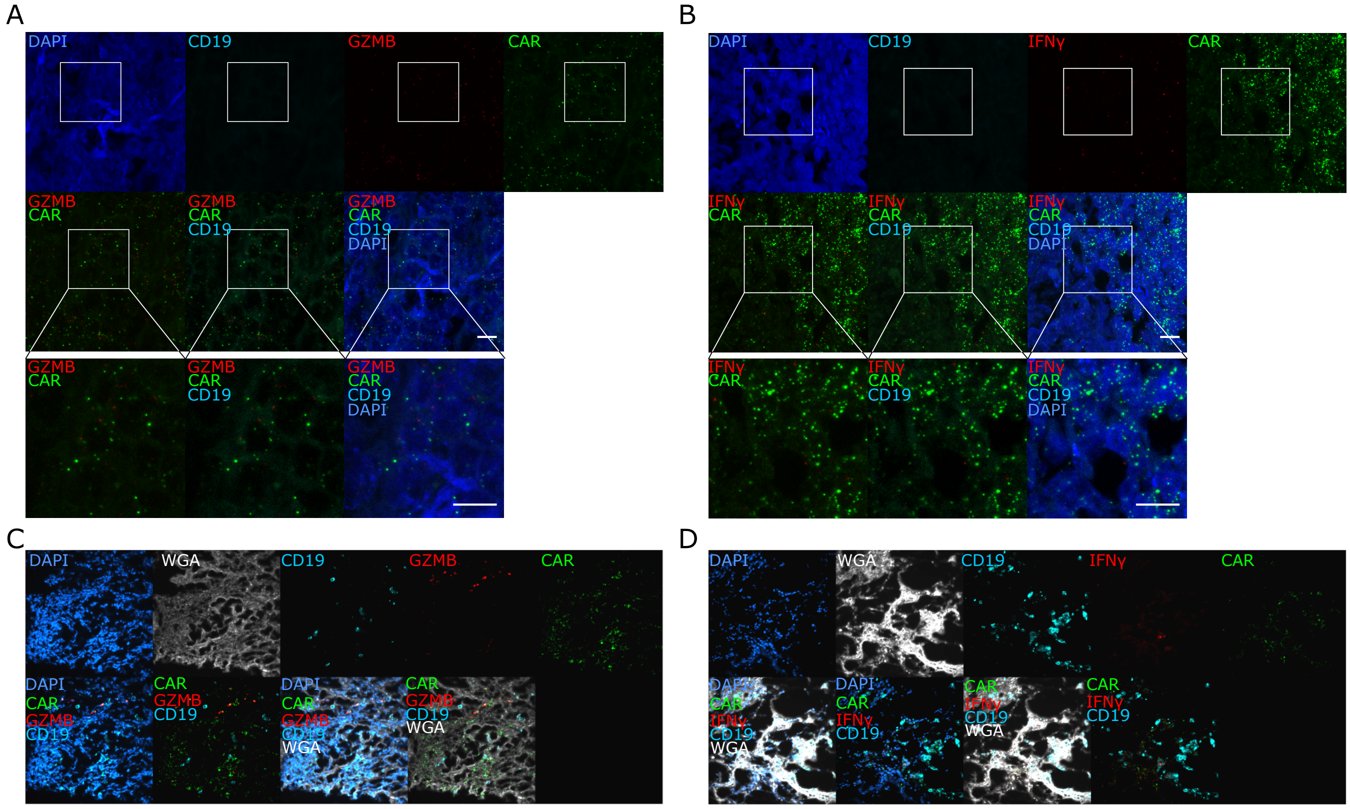

Supplementary Figure 7

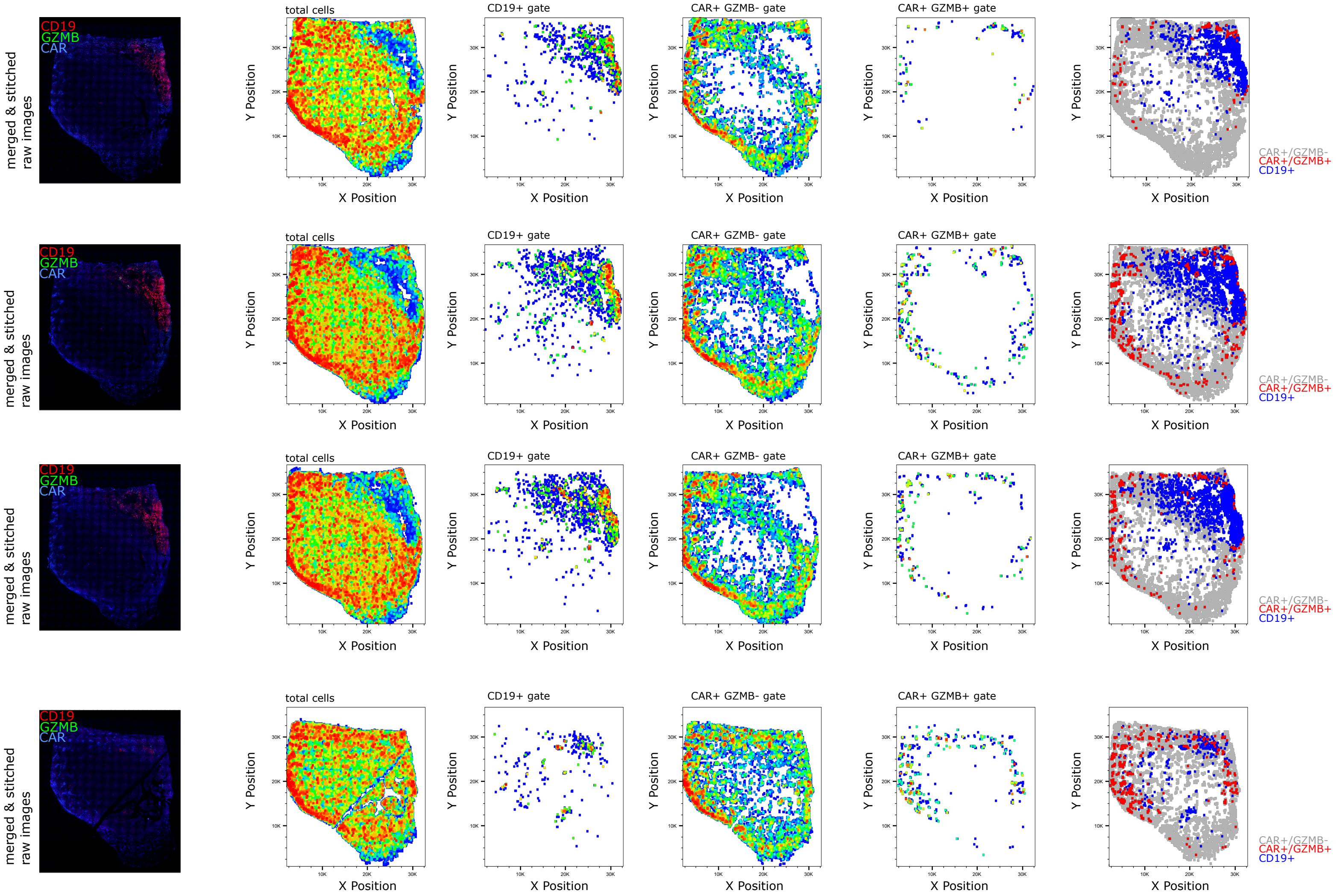

Supplementary Figure 8

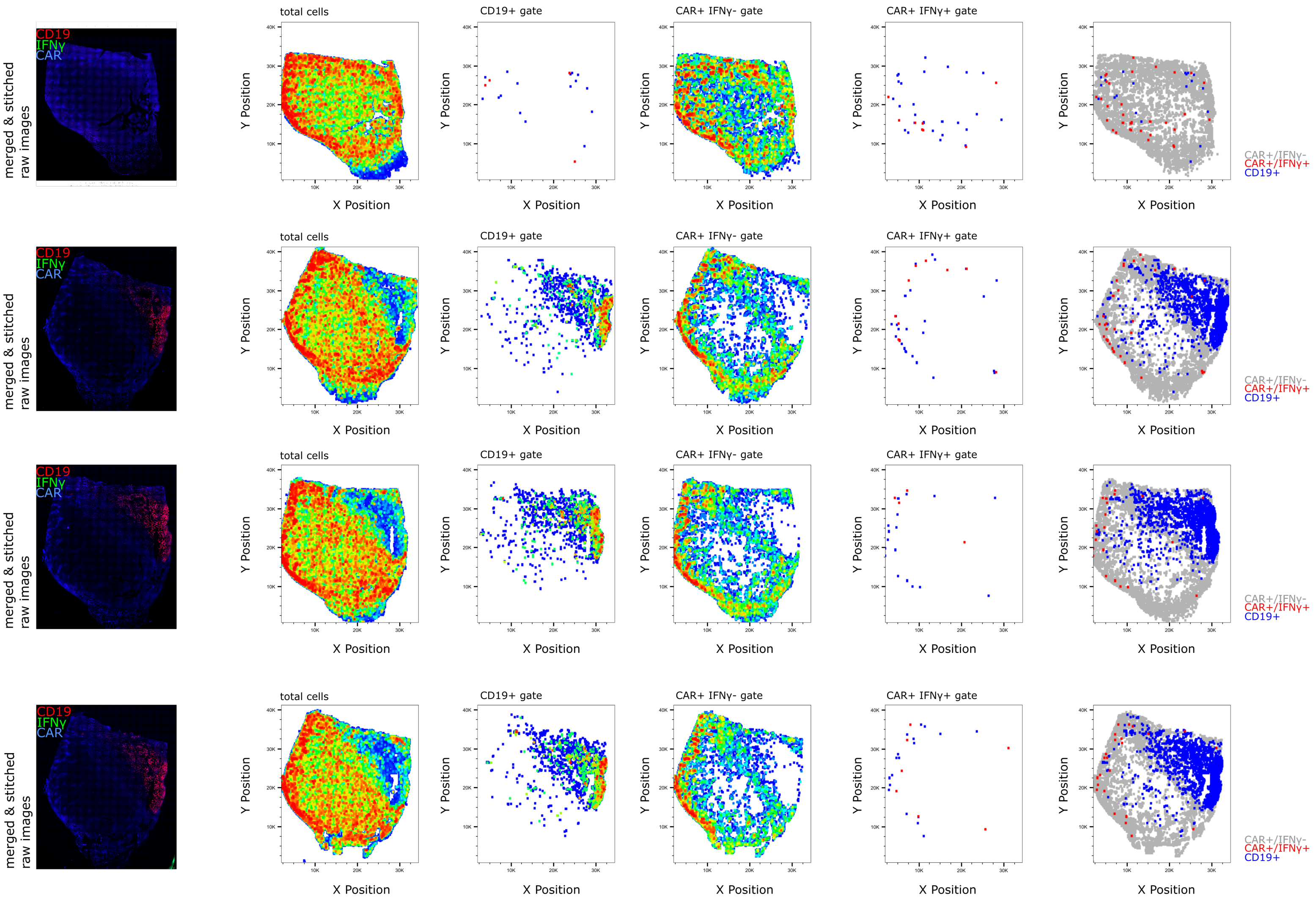

Supplementary Figure 9
